# Essential Oils as an Alternative to Pyrethroids’ Resistance against *Anopheles* Species Complex Giles (Diptera: Culicidae)

**DOI:** 10.3390/molecules22101321

**Published:** 2017-09-22

**Authors:** Olivier Gnankiné, Imaël Henri Nestor Bassolé

**Affiliations:** 1 Laboratoire d’entomologie fondamentale et appliquée (Lefa), Université Ouaga I Pr Joseph KI-ZERBO, 03 P.O. 7021 Ouagadougou, Burkina Faso; olignankine@gmail.com or olivier.gnankine@univ-ouaga.bf; 2 Laboratoire de biologie moléculaire, d’épidémiologie et de surveillance des bactéries et virus transmis par les aliments (Labesta), Université Ouaga I Pr Joseph KI-ZERBO, 03 P.O. 7021 Ouagadougou, Burkina Faso

**Keywords:** essential oils, *Anopheles* sp., insecticides, repellent, pyrethroid resistance

## Abstract

Widespread resistance of *Anopheles* sp. populations to pyrethroid insecticides has led to the search for sustainable alternatives in the plant kingdom. Among many botanicals, there is great interest in essential oils and their constituents. Many researchers have explored essential oils (EOs) to determine their toxicity and identify repellent molecules that are effective against *Anopheles* populations. Essential oils are volatile and fragrant substances with an oily consistency typically produced by plants. They contain a variety of volatile molecules such as terpenes and terpenoids, phenol-derived aromatic components and aliphatic components at quite different concentrations with a significant insecticide potential, essentially as ovicidal, larvicidal, adulticidal, repellency, antifeedant, growth and reproduction inhibitors. The current review provides a summary of chemical composition of EOs, their toxicity at different developmental stages (eggs, larvae and adults), their repellent effects against *Anopheles* populations, for which there is little information available until now. An overview of antagonist and synergistic phenomena between secondary metabolites, the mode of action as well as microencapsulation technologies are also given in this review. Finally, the potential use of EOs as an alternative to current insecticides has been discussed.

## 1. Introduction

Malaria, caused mainly by *Anopheles* mosquitoes, remains a major concern despite many efforts undertaken in vector control strategies. About 90% of malaria deaths worldwide in 2015 were registered in African countries, including the death of a child under five years old every 2 min [1].

Vector control relies primarily on two interventions in the form of long-lasting insecticidal nets (LLINs) and indoor residual spraying (IRS) [2,3]. This combination has saved thousands of lives during the past 10 years [2]. Pyrethroids, organochlorines (dichlorodiphenyltrichloroethane, DDT), organophosphates and carbamates are the active insecticidal ingredients recommended by World Health Organization (WHO) for IRS, while pyrethroids were the only products used for LLINs. These insecticides are known to be neurotoxic. Unfortunately, resistance to most pyrethroids used against adult mosquito populations in public health treatments has been detected in many countries and is now widespread and reported in two thirds of the countries with ongoing malaria transmission problems [4,5,6,7]. 

According to Silva et al. [8], pyrethroid resistance genes, termed as “*knock down resistance*” (*kdr*) have already been found in at least 13 species (*Anopheles gambiae*, *An. arabiensis*, *An. sinensis*, *An. stephensi*, *An. subpictus*, *An. sacharovi*, *An. culicifacies*, *An. sundaicus*, *An. aconitus*, *An. vagus*, *An. paraliae*, *An. peditaeniatus* and *An. albimanus*) from mosquito populations on the African, Asian and, more recently, American continents. Seven mutational variants (L1014F, L1014S, L1014C, L1014W, N1013S, N1575Y and V1010L) have been described, with the highest prevalence of L1014F, which occurs at the 1014 site in channel sodium IIS6 domain [8]. In West Africa, pyrethroid resistance in *Anopheles* mosquitoes is mainly attributed to mutations of the sodium channel target site, the L1014F kdr [8,9,10,11,12,13,14,15,16]. To date, three mutations L1014F known as *kdr*-*West*, L1014S as *kdr*-*East* and N1575Y coexist in some countries and are widely distributed throughout Benin, Cameroon and Burkina Faso [7,8,17,18,19,20,21]. Up to now, in most countries, the *kdr* genes are almost fixed in mosquito populations due mainly to high selection pressure exerted on adults directly but also indirectly in case of pest control in agriculture. It is important to know that chemicals currently used in control of agricultural pests are also the same ones used for vector control, therefore they are source of increasing the potential for resistance selection in mosquitoes as in *An. gambiae* [6].

Human health risks associated with the use of chemicals have led to the growth of an environmental movement seeking sustainable alternatives in pest control. Therefore, in recent years, various workers have been concentrating their efforts on the search for natural products derived from plants as an alternative to conventional insecticides used in controlling vectors for which resistance was detected [22]. Among many natural products, essential oils (EOs) and their constituents have received considerable attention in the search for new pesticides, and have been found to possess an insecticidal potential [23]. These natural compounds are generally recognized as safe (GRAS) for the environment and human health, which explains our interest in their use for a sustainable agriculture and human health.

According to Isman [24], most essential oils (EOs) and their major constituents are relatively non-toxic to mammals, with acute oral Lethal Dose (LD) values in rodents ranging from 800 to 3000 mg·kg^−1^ for pure compounds and >>5000 mg·kg^−1^ for formulated products. Many EOs show toxic effects against several insect species due to their multiple modes and sites of action in the insects’ nervous system [25,26]. Most widely known plants used for protection against mosquitoes belong to the families *Myrtaceae*, *Lauraceae*, *Rutaceae*, *Lamiaceae*, *Asteraceae*, *Apiaceae*, *Cupressaceae*, *Poaceae*, *Zingeberaceae* and *Piperaceae* [25]. According to Nerio [27], it is important to test some parameters of EOs such as their human toxicity, before promoting their use. Although some of them such as citronella, lemon and eucalyptus oils are recommended by the U.S. Environmental Protection Agency (US EPA) as repellent ingredients for application on the skin due to their relative low toxicity, comparable efficiency and customer approval, others might possess a higher toxicity than chemicals and thus cause skin irritation [28].

Essential oils are oily aromatic liquids extracted from plants [29]. Techniques commonly employed for their extraction include hydrodistillation, steam distillation, solvent extraction, head space analysis and liquid CO_2_ extraction [30,31,32,33].

The review focuses on the examination of the recent knowledge concerning the use of essential oils and their constituents against *Anopheles* mosquitoes, the main vector of malaria diseases, for which little information is available. Bioactive components’ interactions, their mode of action and microencapsulation technologies are also addressed in this survey.

## 2. Chemical Composition

Essential oils are natural, complex, multi-component mixture including hydrocarbons (terpenes), oxygenated hydrocarbons (terpenoids), and phenylpropenes [34]. Both terpenes and terpenoids are based on the 2-methylbuta-1,3-diene (C_5_H_8_) unit called isoprene.

**Figure 1:**
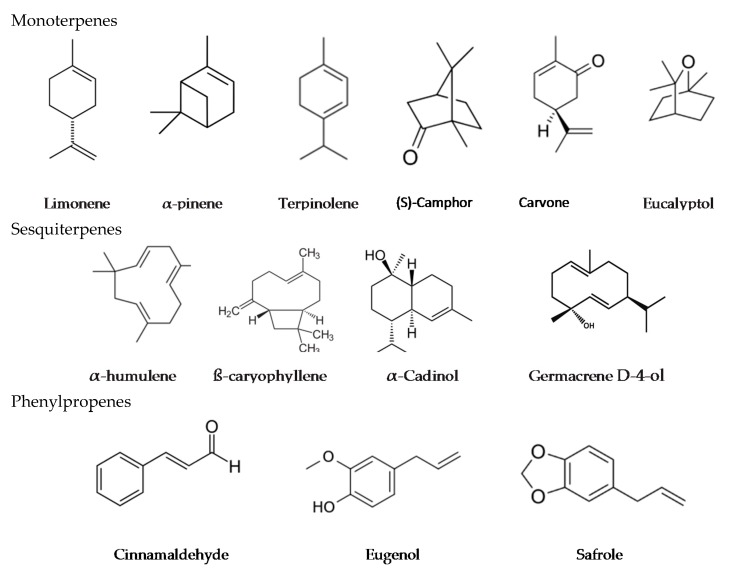
Structural formulae of selected essential oil components.

The isoprene unit builds up in repeating units to form monoterpenes, sesquiterpenes, diterpenes, triterpenes and tetraterpenes containing 2, 3, 4, 6 and 8 isoprene units, respectively. Only monoterpenes, sesquiterpenes and diterpenes are found in essential oils. Monoterpenes together with sesquiterpenes are the most abundant constituents of essential oils [35]. They are either acyclic or cyclic. Terpenoids are terpenes with alcohol, phenol, aldehyde, ketone or ester functional groups (Figure 1). Phenylpropenes are compounds based on a phenylpropane skeleton and many are derived from a biochemical pathway called the shikimic acid pathway.

## 3. Toxicity to Eggs and Immature Stages in *Anopheles* sp.

Many plant essential oils are reported to possess ovicidal, larvicidal and pupacidal activities against malaria vectors according to the WHO test procedures [36,37,38,39]. Research reports show that essential oils have ovicidal activities against *Anopheles gambiae* s.l., *An. gambiae* s.s. [37], and *An. stephensi* (Table 1) [36,37].

Bassolé et al. [37] tested the efficiency of *Cymbopogon proximus*, *Lippia multiflora* and *Ocimum canum* essential oils against *An. gambiae* eggs and found that the LC_50_ values ranged from 61.9 to 188.8 mg·L^−1^. Eggs of *An. stephensi* were susceptible to the essential oils of *Cinnamomum zeylanicum*, *Cuminum cyminum*, *Curcuma longa*, *Juniperus macropoda*, *Ocimum basilicum*, *Pimpinella anisum*, *Zingiber officinalis* and *Cinnamomum zeylanicum*. LC_95_ varied from 32.2 to 172.8 mg·L^−1^ [36].

According to Dias and Moraes [75], plant essential oils were considered active when their LC_50_ values were below 100 mg·L^−1^ against larvae vector mosquitoes such as *Aedes* sp. Thus, essential oils from 63 plant species demonstrated larvicidal activity against *Anopheles anthropophagus*, *An. atroparvus*, *An. arabiensis*, *An. dirus*, *An. funestus*, *An. gambiae* s.l., *An. gambiae* s.s., *An. quadrimaculatus*, *An. stephensis* and *An. subpictus* (Table 2). Table 2 shows that LC_50_ values were between 1.8 and 91.2 mg·L^−1^ whereas LC_90_ were from 4.1 to 199 mg·L^−1^. Many researchers have shown the efficiency of major compounds from EOs on *Anopheles* sp. larvae (Table 3). Essential oil from *Curcuma longa* was the most toxic against *An. quadrimaculatus* larva. All values are above the World Health Organization (WHO) guideline of 1 mg·L^−1^ for larvicides. The use of EOs extracted from plants seems to be problematic with regards to their LC_50_ values. However, these studies should be interesting, if the tests carried out take into account the susceptibility of reducing the use of the chemicals commonly used in human protection.

## 4. Essential Oils Toxicity in *Anopheles* sp. Adults

In addition to their effects on eggs and larvae, EOs exhibit toxic effects on adults of malaria vectors following WHO test kits and topical applications recommended by WHO [36,39,76,77] (Table 4). Recently, Deletre et al. [39] tested the toxic effects of 20 plant extracts on the adults of the malaria vector *An. gambiae*. Amongst them, the most promising plant extracts are those from *Cymbopogon winterianus*, *Cinnamomum zeylanicum* and *Thymus vulgaris*. The mortality due to these EOs was higher than that observed with permethrin. In Benin, Bossou et al. [77] investigated on the effect of nine EOs extracted from plants on susceptible strain “Kisumu”. The plants tested were *Chenopodium ambrosioides* L., *Securidaca longepedunculata Fresen.*, *Cochlospermum planchonii* Hook. f. Ex Planch., *Cochlospermum tinctorium* A. Rich., *Eucalyptus tereticornis* Sm., *Eucalyptus citriodora* Hook. *Cymbopogon citratus* (DC.) Stapf, *Cymbopogon schoenanthus* (L.) Spreng. and *Cymbopogon giganteus* Chiov. They showed that mortality rate of *An. gambiae* “Kisumu” varies depending on the concentration. The most efficient essential oil was *C. citratus*, followed by *E. tereticornis*, *E. citriodora* and *C. ambrosioides*, *C. schoenanthus*, *C. giganteus*, *C. planchonii* and *S. longepedunculata* (Table 4). These oils showed 100% mortality rates. For instance, the threshold of susceptibility was fixed at 98% for the active molecules according to the WHO protocol [78]. The resistance/susceptibility status was evaluated according to WHO [78] criteria, considering mortality above 97% and below 90% representative of susceptibility and resistance, respectively, but between the two values, resistance should be suspected. 

*An. gambiae* resistant strains were susceptible to all essential oils at the diagnostic doses tested, except for *C. tinctorium* and *S. longepedunculata*, for which resistance was suspected because the mortality was less than 97%. Then, according to the WHO protocols [78], diagnostic doses are defined as twice the lethal concentration (LC) for 99% mortality (LC_99_) on sensitive strains.

According to Bossou et al. [77], *C. citratus*, *E. tereticornis*, *E. citriodora*, *C. ambrosioides* and *C. schoenanthus* are potential promising plant sources for alternative compounds to pyrethroids for the control of the *Anopheles* malaria vector in Benin, where pyrethroid resistance was detected in the south part of the country, due to the massive use of permethrin and DDT. On the contrary, with the topical application, permethrin and deltamethrin seem to be toxic than the EOs in *An. gambiae* populations tested in a study done by Norris [76]. 

## 5. Repellency Effects of EOs against *Anopheles* Mosquito Adults

The repellent ability of EOs seems to be the major promising entomological use in the human health. According to Choochote [79], repellents are substances that act locally or at a distance, deterring an arthropod from flying, landing on or biting humans or animals (or any surface in general). It is also defined as a phenomenon that prevents a pest’s ability to track, locate and/or recognize its host. Hence, a repellent phenomenon can be a movement away from an odor source, but also an inability to find the host [80]. Recently, a review on repellency effects on diseases vectors has been published [25]. This review reported current evaluation approach of EOs and various methodology of repellents effects of synergists of EOs constituents. 

In terms of methodology, the human bait method is mainly used as a common procedure. However, studies including positive controls are very scarce. In 2016, the review of Deletre et al. [80] identified five (5) types of repellent on the basis of the observed insect behavior: (i) true repellent (also called expellent, spatial repellent), which corresponds to an oriented movement of the insect away from an odor source without direct contact, (ii) odor masking (also called attraction inhibition), which is either a reduction in the attractiveness of the host or a disruption of the localization of the host by the odor cue, (iii) contact irritancy (also called landing inhibition or excito-repellent), an oriented movement of the insect away from a chemical after direct contact, (iv) deterrence (also called antifeeding, suppressant, anorexigenic and anti-appetant), which corresponds to a disruption of feeding activity by contact with or ingestion of a chemical, and (v) visual masking, which defines a reduction in the attractiveness of the host or a disruption of the localization of the host by a visual cue.

Amer and Mehlhorn [81] tested essential oils from 41 plants against *An. stephensi* using the human-bait technique [82]. The twelve most effective oils were those of *Nepeta cataria*, *Jasminum grandiflorum*, *Cymbopogon citratus*, *Cinnamomum zeylanicum*, *Melaleuca leucadendron*, *Amyris balsamifera*, *Melaleuca quinquenervia*, *Tagetes minuta*, *Viola odorata*, *Santalum album*, *Litsea cubeba* and *Ferula galbaniflua* which induced a maximum protection time of 8 h and showed repellency percentage of 100% as that of DEET (The positive control). By using the same technique, Rajkumar and Jebanesan [83] evaluated EO of *Centella asiatica* L., *Ipomoea cairica* L., *Momordica charantia* L., *Psidium guajava* L. and *Tridax procumbens* L. against *An. stephensi*. The EOs of *I. cairica*, *M. charantia* and *T. procumbens* at 6% concentration exhibited relatively high repellency effect (>5 h), followed by *C. asiatica* and *P. guajava* which showed less effective (<2.5 h) [83]. Phasomkusolsil and Soonwera [84], tested the essential oils of *Cymbopogon citratus*, *Cymbopogon nardus*, *Cananga odorata*, *Citrus sinensis*, *Eucalyptus citriodora*, *Ocimum basilicum* and *Syzygium aromaticum* against Anopheles dirus at 0.21 mg/cm^2^. The repellency percentages were 84% to 98% and protection time varied from 24 to 132 min (Table 5).

In addition to their high toxicity against *Anopheles* sp. adults, the EOs from *Cymbopogon winterianus*, *Cuminum cyminum*, *Cinnamomum zeylanicum* and *Thymus vulgaris* possessed repellency and irritant actions by using treated paper technology recommended by the WHO [39]. In the repellent tests and at 1%, the proportion of mosquitoes escaping was above those found when permethrin and DEET was used as the positive controls [39]. Also, these EOs were irritants as well as DEET and permethrin (positive controls).

Abagli and Alavo [85] showed the potential of *H. suaveolens* EO as a mosquito repellent by the human–bait technique. After 6 h application, the mean number of *An. gambiae* that landed on treated volunteers was 0.50 and 0.45 for 10% *H. suaveolens* essential oil and DEET respectively, against six mosquitoes for the control subjects. *H. suaveolens* essential oil and DEET possess similarly effects.

## 6. Biologically Active Components of Essential Oils

The insecticidal activity of EOs is dependent on their chemical composition and on interactions between individual compounds [86]. Studies reported that EO activity is the result of their inherent biologically active components (Table 3 and Table 6). Generally, EO showing higher efficiency against larvae and adults mosquitoes was dominated by monoterpenes (Table 6). 

The larvicidal activities of the essential oil can be related to its main compounds. The three main components of the essential oil of *Mentha spicata*—carvone, *cis*-carveol, and limonene—were responsible of its larvicidal activity against *An. Stephensi* [87]. Also, α-humulene and β-elemene, two main components of the essential oil of *Syzygium zeylanicum*, appeared highly effective against *An. subpictus* with LC_50_ values of 6.19 and 10.26 μg/mL, respectively against 83.11 μg/mL for the whole essential oil [65]. Ar-turmerone, a major component of *Curcuma longa* leaf and rhizome essential oils showed higher and lower efficiency against larvae of *An. quadrimaculatus* than that of the whole oil from *Curcuma longa* rhizome and leaf, respectively [50].

Carvacrol and terpinen-4-ol, two major constituents extracted from the *Ocimum vulgare* EO appeared to be most effective against *An. stephensi* (LC_50_ = 21.15 and 43.27 µg/mL, respectively) and *An. subpictus* larvae (LC = 24.06 and 47.73 µg/mL, respectively) [88]. The three main components of the essential oil of *Plectranthus Barbatus*—eugenol, α-pinene and β-caryophyllene—exerted the most potent larvicidal activity against *An. subpictus* (LC_50_ = 25.45, 32.09 and 41.66 μg/mL, respectively) [63]. 

The minor compounds could be also implicated in the adult mosquito toxicity of an essential oil. Thus, the toxicity of *C. winterianus* and *C. cymimum* oils could be due to their minor compounds, whereas this was not the case for *C. zeylanicum* and *T. vulgaris* oils, which toxicity could be due to cinnamaldehyde and the major compounds in the blend [90]. α-Pinene, limonene, terpinolene, citronellol, citronellal, camphor and thymol are common constituents of a number of essential oils that show mosquito repellent activity [91,92,93,94,95]. Among the sesquiterpenes, β-caryophyllene is considered to be a strong repellent against *Anopheles sp*. [91,96,97]. Phytol, a linear diterpene alcohol, possessed high repellent activity against *An. gambiae* in Kenya [98]. Repellent and irritant effects of *Thymus vulgaris*, *Cuminum cymimum* and *Cinnamomum zeylanicum* essential oils are usually considered due to one major compound [90]. Thus, the repellency effects of *T. vulgaris*, *Cymbopogon winterianus* and *Cuminum cymimum* was attributed to their main components carvacrol, citronellal, geraniol and cuminaldehyde, respectively [90]. Moreover, thymol and/or carvacrol; citronellal, geraniol, and/or citronellol; cuminaldehyde; and cinnamaldehyde, respectively, are implicated in *T. vulgaris*, *C. winterianus*, *C. cymimum* and *C. zeylanicum* oils’ irritant effects. In fact, there was no significant difference between the single compounds and associated essential oils [90]. 

## 7. Synergistic and Antagonistic Phenomena

The inherent activity of an EO can be expected to be related to the chemical configuration of the components, the proportions in which they are present and to interactions between them [99]. An additive effect is observed when the combined effects are equal to the sum of the individual effects. Antagonism is observed when the effects of one or both compounds are less when they are applied together than when applied alone. Synergism is observed when the effect of the combined substances is greater than the sum of the individual effects [100]. In some cases, the whole essential oil exhibited a greater pesticidal activity than its major components isolated, suggesting that the minor components are critical to the activity and may have a synergistic effect or potentiating influence [101].

In general, it appears that the effect of an active compound could be enhanced by other major compounds and/or modulated by minor compounds to give additive or synergistic effects. For example, repellency and toxicity of (*E*)-cinnamaldehyde could be synergistically enhanced by minor compounds like benzaldehyde, coumarin, phenyl ethyl alcohol, and (*Z*)-cinnamaldehyde, while the irritancy of carvacrol appeared to be reduced by minor compounds such as myrcene, borneol, α-pinene, γ-terpinene, terpinen-4-ol, limonene, and α-thujene [90]. Accordingly, minor constituents found in low percentages may act as synergists, enhancing the effectiveness of the major constituents through a variety of mechanisms [102]. A synergistic phenomenon among the metabolites may result in a higher bioactivity compared to the isolated components [97]. This synergistic effect is also observed with mixtures of oils. The high insect repellency and toxicity of the mixed oils might have resulted from synergistic action of the main components in the oils.

Studies on the interactions between oil compounds against *An. gambiae* remain very scarce. Recently, Deletre [90] investigated and showed a complexity in the biological activity of EOs for toxicity, irritancy and repellent tests. Nevertheless, no correlation was found between the activity of EO and activity from their major components. Many of the repellent studies have shown that synthetic compounds or blends of pure compounds are less effective when compared to the activity of their corresponding essential oils [25]. That was the case of cinnamaldehyde, the major constituent of *C. zeylanicum* [90].

In terms of toxicity, no synergistic effect was observed between the oil components but an antagonist effect was observed between a Cinnamaldehyde, a major constituent of *C. zelanicum* and other components explaining their low value [90]. An antagonistic effect between cuminaldehyde (a major component) and another constituent will be suspected for *Cuminum cyminum* EO due to the fact that the cumin blend was less irritant than essential oil.

## 8. Mechanisms of Action of Essential Oil Components

The modes of action of EOs are well documented [103], but nevertheless this review will highlight the target sites of EOs. Most monoterpenes are toxic to insects by penetrating the body through the respiratory system (fumigant effect), the cuticle (contact effect) or through the digestive system in case of ingestion [104,105].Several monoterpenes present in EOs are neurotoxic to insects. Rattan [106] described three major targets of insect neurosystems (the cholinergic, octopamenergic and GABA systems).

Acetylcholinesterase (AChE) plays a key role in cholinergic synapses that are essential for insects and higher animals [107]. It is known to be a class of enzymes that catalyzes the hydrolysis of the neurotransmitting agent acetylcholine (ACh). Inhibition of AChE causes accumulation of acetylcholine in the synapses, so that the post-synaptic membrane is in a state of permanent stimulation, which results in ataxia i.e., general lack of co-ordination in the neuromuscular system, and eventual death [103,108]. A number of monoterpenes also act on acetylcholinesterase [103,109]. For example, linalool a monoterpene that is the major compound of *C. sativum* essential oil, showed a toxic effect to mosquitoes and was identified as an inhibitor of acetylcholinesterase [105].

According to Abdelgaleil et al. [110] monoterpenes such as cuminaldehyde, 1,8-cineole, limonene and fenchone have strong insecticidal activity and potent AChE inhibitory activity whereas other compounds like geraniol or *R*-carvone have strong insecticidal activity but are weak inhibitors of AChE. Hideyuki and Mitsuo [111] noted that the mixture of monoterpenoids could be active as synergists in the inhibition of AChE. In fact, these authors found out that limonene, linalool and lynalyl acetate mixture showed synergistic effects and were more inhibitory than bergamot oil or single monoterpenoid applications.

Carvacrol, α-pinene, and β-pinene inhibited the activity of *Aedes albopictus* larvae acetylcholinesterase with IC values of 0.05, 0.06, and 0.19 mg/mL, respectively [112]. Savelev et al. [113] by analysing synergistic and antagonistic interactions of anticholinesterase terpenoids, concluded with the evidence of synergy since the inhibitory activity of individual terpenes was lower than the whole oil. Keane and Ryan [114] mentioned that the validation of an inhibition in vitro of the AChE should be demonstrated additionally by an appropriate effect in vivo. These authors also pointed out the importance of not excluding additional modes of action of monoterpenoids.

Another possible target for essential oil activity is the octopaminergic system of insects. Octopamine is a naturally occurring multifunctional biogenic amine, which plays key roles as a neurotransmitter, neuromodulator and neurohormone in invertebrate systems, with a physiological function similar to that of nornephrine in vertebrates [115]. The acute and sub-lethal behavioral effects of essential oil compounds on insects and other vertebrates are consistent with an octopamenergic target site in insects, which acts by blocking octopamine receptors [116,117].

Physiological functions of OA appears to be mediated by pharmacologically distinct class of octopamine receptors viz. octopamine receptors (myogenic rhythm via Caþ concentration), octopamine 2A and octopamine 2B, octopamine 3 (activation of adenylate cyclise activity), which are coupled to different second messenger system belonging to the family of G-protein coupled receptors (G-PCRs) [117,118,119,120]. It was subsequently shown that treatment with the octopaminergic antagonist phentolamine effectively inhibited the cyclic AMP levels induced by essential oil treatment, indicating a possible competitive activation of octopaminergic receptors by essential oil constituents.

Enan [117] showed that low doses of eugenol caused a significant increase in adenosine 3′,5-cyclic monophosphate (cAMP) in the nervous system of *P. americana*, a similar effect to that of octopamine. The increase in cAMP caused by octopamine was blocked by a mixture of three essential oil constituents: eugenol, γ-terpineol and cinnamic alcohol. It was also demonstrated that low doses of eugenol significantly decreased octopamine receptor binding. 

Ligand-gated chloride channels are recognized to be a target site for insecticides acting as antagonists by stabilizing non-conducting conformations of the chloride channel. Blockage of the GABA-gated chloride channel reduces neuronal inhibition, which leads to hyper-excitation of the central nervous system, convulsions, and then death [121]. 

Based on the [^3^H]-TBOB binding assay and ^36^Cl^−^ uptake assay, Tong and Coats [122] showed that some monoterpenoids can affect the functioning of insects’ GABA systems by binding to the GABA receptor and increasing the chloride uptake activated by GABA. Their studies revealed that in the American cockroach’s ventral nerve cord, carvacrol, pulegone, and thymol all significantly increased the ^36^Cl^−^ uptake stimulated by GABA, and that they did not increase the ^36^Cl^−^ uptake in the absence of GABA in the assay system showing that carvacrol, pulegone, and thymol were all positive allosteric modulators for insect’s GABA receptor. They modulated the insect GABA system by binding at the receptor and increasing chloride anion influx into the neurons. 

Thujone is also classified as a neurotoxic insecticide, which acts on GABA receptors [123,124]. Thujone is a competitive inhibitor of [^3^H] EBOB binding (i.e., of the non-competitive blocker site of the GABA-gated chloride channel) and is a reversible modulator of the GABA receptor. It was suggested that thymol potentiates GABA receptors through an unidentified binding site [125].

## 9. Microencapsulation and Nanoemulsion Technologies

Application of essential oils is limited due to their rapid volatility. However, with new currently available technologies, it is possible to improve the duration of action of EOs. Microencapsulation is a technology for packaging an active ingredient in the form of droplets of a solid or liquid material (the core) inside a capsule, that is, a continuous film of polymeric material (the shell) ranging in size from one micron to several millimeters [126].

Microencapsulation processes are categorized into two groups: chemical processes and mechanical or physical processes [126]. The morphology of microcapsules can be as follows: (i) mononuclear containing the shell around the core; (ii) polynuclear that have many cores enclosed within the shell or (iii) matrix with the core material is distributed homogenously in the shell material [126]. Commercial microcapsules usually have a diameter between 3 and 800 μm and contain 10–90 wt % core [127]. 

The active products of EOs can be encapsulated, used with polymer resins or synergized by other compound like vanillin [105,128]. According to Tawatsin [129], oils from *Curcuma longa*, *Cymbopogon winterianus* and *Ocimum americanum*, especially with the addition of 5% vanillin, repelled *Anopheles dirus* under cage conditions for up to eight hours.

The compounds should be selected taking into account to their low toxicity to humans and must provide protection for at least 4 h. According to Deletre [90], compounds from citronella can be potential alternatives to repellents, especially since they are non-toxic individually and when mixed. This explains why this EO is used in microencapsulation experiments. Recently, a simple, low cost, scalable, and reproducible method has been set up by Specos [130]. This technology consists in preparing complex of coacervation microcapsules containing citronella EO. Using this technology, the study aimed at assessing repellent activity by exposure of a human hand and arm covered with treated textiles to *Aedes aegypti* mosquitoes. According to these authors, fabrics treated with microencapsulated citronella exhibited a higher and longer lasting protection from insects compared to fabrics sprayed with an ethanol solution of the essential oil, assuring a repellent effect higher than 90% for up to three weeks. Salomon et al. [131] showed that microencapsulation decreased membrane permeation of citronella EO by at least 50%. Another technology is encapsulated citronella oil nanoemulsion which is prepared by high pressure homogenization at varying amounts of surfactant and glycerol [132]. These authors showed that the release of citronella oil from high amounts of glycerol was much slower than that from the low glycerol amounts resulting in sustained mosquito protection time.

## 10. Concluding Remarks

Pyrethroids are widely used in controlling mosquitoes. They are used in bednet treatments, impregnation of clothes, indoor residual spraying and spatial treatments [133]. The advantage of pyrethroids is their effectiveness at low dosages. They are also toxic, irritant, fast acting, stable and safe for humans [134,135]. According to Duvallet [133], pyrethroids have four main effects on mosquitoes causing: (i) a spatial repellent effect by deterrence of adults from entering treated rooms; (ii) a contact irritant effect by short-lived settling of mosquitoes on treated bednets or walls; (iii) an anti-feedant effect by inhibition of blood feeding by female mosquitoes and (iv) toxic effect by inducing a knock down (KD) and mortality effect. In Africa, pyrethroid resistance in *Anopheles* populations is now widespread and could compromise the vector control strategies. Thus, the use of EOs as mosquito control insecticides would be greatly increased if their mode of action is not the same as that of currently used chemical products [136]. This raises the following question: Can EOs be used as an alternative to the pyrethroid resistance?

A number of EOs and several of their individual components exhibit insecticidal activity against several disease vectors. Their mechanism of action depends on component involving several targets such as the cholinergic, octopamenergic and GABA systems [137]. 

Therefore, knowledge of the physiological mode of action of these active essential oils is of great importance for their future use in health. EOs might be used as alternative to the resistance of pyrethroids if it were demonstrated that only channel sodium voltage dependant target sites are involved in this resistance. In fact, many EOs or their components act as inhibitors of acetylcholinesterase while the sodium channel voltage dependent is mainly implicated in pyrethroid resistance.

However, some cases of resistance to organophosphates and carbamates are reported due to the mutation in acetylcholinesterase gene 1 (*ace-1^R^*) [5,6,7], with a percentage reaching rarely 30% up till now [138]. Fortunately, this rate may decrease in the absence of OP and carbamate applications. Then, homozygous and heterozygous *ace-1^R^* vectors, which survive in the presence of insecticide, may be rapidly outcompeted in the absence of insecticide.

The synergistic interactions between EOs components are interesting; it is possible to mix two or three compounds with different effects to avoid habituation behaviour from mosquitoes. To date, microencapsulation, which improves the duration of action of certain EOs, can favor the use of ingredients of EOs in terms of impregnation of mosquito bednets. In conclusion, EOs have both toxic and repellent actions and could be envisaged as a way for sustainable management of pyrethroid resistance in Africa.

## Figures and Tables

**Table 1:** Toxicity of essential oils against eggs of *Anopheles* sp.

Plant Species	Plant Organ	Extraction Method	Species	LC_50_ (mg/L)	LC_90_ (mg/L)	LC_95_ (mg/L)	Reference
*Cinnamomum zeylanicum*	*Bark*	Hydrod.	*An. stephensi*	NI	NI	130.0	[36]
*Cuminum cyminum*	Seed	Hydrod.	*An. stephensi*	NI	NI	150.7	[36]
*Curcuma longa*	Rhizome	Hydrod.	*An. stephensi*	NI	NI	89.9	[36]
*Juniperus macropoda*	Fruit	Hydrod.	*An. stephensi*	NI	NI	59.3	[36]
*Ocimum basilicum*	Leaf	Hydrod.	*An. stephensi*	NI	NI	172.8	[36]
*Pimpinella anisum*	Seed	Hydrod.	*An. stephensi*	NI	NI	33.3	[36]
*Zingiber officinalis*	Rhizome	Hydrod.	*An. stephensi*	NI	NI	32.2	[36]
*Lippia multiflora*	Leaf	Hydrod.	*An. gambiae* s.s	17.1	33.5	NI	[37]
*Ocimum canum*	Leaf	Hydrod.	*An. gambiae* s.s	188.7	488.0	NI	[37]
*Cymbopogon proximus*	Leaf	Hydrod.	*An. gambiae* s.s	52.8	91.1	NI	[37]

Hydrod. = Hydrodistillation; LC_50_, LC_90_ and LC_95_ are the lethal concentrations that kill 50%, 90% and 95% of eggs, respectively; NI: No information is available.

**Table 2:** Toxicity of essential oils against larvae of *Anopheles* sp.

Plant Species	Plant Organ	Extraction Method	Mosquito	LC_50_ (mg/L)	LC_90_ (mg/L)	LC_95_ (mg/L)	LC_99_ (mg/L)	Ref.
*Amomum subulatum*	Leaf	Hydrod.	*An. subpictus*	41.2	80.3	NI	NI	[40]
*Apium graveolens*	Whole plant	Steam-d.	*An. dirus*	59.4		111.7	159.1	[41]
*Artemisia gilvescens*	Plant	Hydrod.	*An. anthropophagus*	49.9	97.4	NI	NI	[42]
*Artemisia gilvescens*	Whole	Hydrod.	*An. anthropophagus*	49.0	97.0	NI	NI	[42]
*Blumea densiflora*	Aerial parts	Hydrod.	*An. anthropophagus*	10.0	33.0	NI	NI	[42]
*Bunium persicum*	Seed	Hydrod.	*An. stephensi*	27.7	91.3	NI	NI	[43]
*Carum carvi*	Whole plant	Steam-d.	*An. dirus*	72.2	NI	104.7	128.7	[41]
*Chenopodium ambrosioides*	Aerial parts	Hydrod.	*An. arabiensis*	17.5	NI	NI	NI	[44]
*Chloroxylon swietenia*	Leaf and stem	Hydrod.	*An. stephensi*	14.0	22.0	NI	NI	[45]
*Cionura erecta*	Root	Hydrod.	*An. stephensi*	77.0	199.0	NI	NI	[46]
*Citrus aurantium*	Fruit	Hydrod.	*A. stephensi*	31.2	73.8	NI	NI	[47]
*Citrus paradisi*	Fruit	Hydrod.	*An. stephensi*	35.7	70.2	NI	NI	[47]
*Coleus aromaticus*	Leaf	Hydrod.	*An. subpictus*	60.3	118.7	NI	NI	[48]
*Cryptomeria japonica*	Leaf	Hydrod.	*An. gambiae*	67.1	193.7	NI	NI	[49]
*Curcuma longa*	Leaf	Hydrod.	*An. quadrimaculatus*	1.8	4.1	NI	NI	[50]
*Curcuma longa*	Rhizome	Hydrod.	*An. quadrimaculatus*	3.7	9.4	NI	NI	[50]
*Curcuma zedoaria*	Whole plant	Steam-d.	*An. dirus*	29.7	NI	40.2	47.7	[41]
*Cymbopogon citratus*	Leaf	Hydrod.	*An. funestus*	34.6	NI	NI	NI	[51]
*Cymbopogon citratus*	Leaf	Hydrod.	*An. gambiae*	18.0	NI	NI	NI	[38]
*Cymbopogon proximus*	Leaf	Hydrod.	*An. gambiae*	69.7	NI	NI	NI	[37]
*Feronia limonia*	Leaf	Hydrod.	*An. stephensi*	15.0	36.7	NI	NI	[52]
*Ferulago carduchorum*	Aerial parts	Hydrod.	*An. stephensi*	12.0	47.0	NI	NI	[53]
*Ferulago carduchorum*	Aerial part	Hydrod.	*An. stephensi*	12.0	47.0	NI	NI	[53]
*Foeniculum vulgare*	Whole plant	Steam-d.	*An. dirus*	35.3	NI	38.8	40.9	[41]
*Juniperus procera*	Leaf	Hydrod.	*An. arabiensis*	24.5	34.2	NI	NI	[54]
*Lavandula gibsoni*	Plant	Hydrod.	*An. stephensi*	62.8	129.0	NI	NI	[55]
*Lippia multiflora*	Leaf	Hydrod.	*An. gambiae*	61.9	NI	NI	NI	[37]
*Mentha spicata*	Aerial part	Hydrod.	*An. stephensi*	49.7	101.0	NI	NI	[56]
*Mentha spicata*	Leaf	Hydrod.	*An. stephensi*	82.9	NI	NI	NI	[57]
*Murraya exotica*	Leaf	Hydrod.	*An. stephensi*	56.3	107.8	NI	NI	[58]
*Nigella sativa*	Leaf	Hydrod.	*An. arabiensis*	23.4	NI	NI	NI	[44]
*Ocimum basilicum*	Leaf	Hydrod.	*An. subpictus*	9.75	18.6	NI	NI	[56]
*Ocimum canum*	Leaf	Hydrod.	*An. funestus*	91.2	NI	NI	NI	[51]
*Ocimum lamiifolium*	Leaf	Hydrod.	*An. arabiensis*	20.9	NI	NI	NI	[44]
*Origanum vulgare*	Leaf	Hydrod.	*An. subpictus*	67.0	128.6	NI	NI	[59]
*Piper capense*	Plant	Hydrod.	*An. gambiae*	34.9	85.0	NI	NI	[60]
*Plectranthus amboinicus*	Leaf	Hydrod.	*An. gambiae*	55.2	99.1	NI	NI	[61]
*Plectranthus amboinicus*	Leaf	Hydrod.	*An. stephensi*	28.37	59.4	NI	NI	[62]
*Plectranthus barbatus*	Leaf	Hydrod.	*An. subpictus*	84.2	165.2	NI	NI	[63]
*Plectranthus mollis*	Whole plant	Hydrod.	*An. stephensi*	33.5	NI	NI	NI	[55]
*Ruta chalepensis*	Aerial parts		*An. quadrimaculatus*	15.0	42.0	NI	NI	[64]
*Salvia elegan*	Aerial parts	Hydrod.	*An. quadrimaculatus*	6.2	15.8	NI	NI	[50]
*Salvia leucantha*	Aerial parts	Hydrod.	*An. quadrimaculatus*	10.9	29.1	NI	NI	[50]
*Salvia officinalis*	Aerial parts	Hydrod.	*An. quadrimaculatus*	14.1	35.8	NI	NI	[50]
*Schinus molle*	Leaf and seed	Hydrod.	*An. arabiensis*	21	NI	NI	NI	[44]
*Syzygium zeylanicum*	Leaf	Hydrod.	*An. subpictus*	83.1	164.2	NI	NI	[65]
*Tagetes patula*	Leaf	Steam-d.	*An. stephensi*	12.0	57.0	NI	NI	[66]
*Toddalia asiatica*	Root	Hydrod.	*An. stephensi*	69.0	110.0	NI	NI	[67]
*Trachyspermum ammi*	Seed	Steam-d.	*An. stephensi*	80.8	NI	NI	172.1	[68]
*Zanthoxylum armatum*	Seed	Hydrod.	*An. stephensi*	58.0	183.0	NI	NI	[69]
*Zanthoxylum limonella*	Whole plant	Steam-d.	*Anopheles dirus*	57.2	NI	76.2	89.5	[41]
*Zhumeria majdae*	Leaf	Hydrod.	*An. stephensi*	61.3	135.8	NI	NI	[70]
*Zingiber nimmoni*	Rhizome	Hydrod.	*An. stephensi*	41.2	80.3	NI	NI	[59]

Hydrod. = Hydrodistillation; Steam-d. = Steam distillation; LC_50_, LC_90_ and LC_95_ are the lethal concentrations that kill 50%, 90% and 95% of eggs, respectively; NI: No information is available.

**Table 3:** Toxicity of essential oils components against *Anopheles* sp. larvae.

Components of EO	Species	LC_50_ (mg/L)	LC_90_ (mg/L)	Reference
**Monoterpene hydrocarbons**				
(+)-Limonene	*An. gambiae* s.s.	270.3	NI	[71]
Limonene	*An. stephensi*	8.8	17.6	[72]
α-Pinene	*An. subpictus*	32.1	62.8	[73]
Terpinolene	*An. gambiae s.s.*	404.71	NI	[71]
Camphor	*An. anthropophagus*	129.7	192.4	[42]
Carvacrol	*An. subpictus*	21.1	41.9	[65]
Carvone	*An. stephensi*	19.3	37.1	[72]
(−)-Carvone epoxide	*Anopheles gambiae* s.s.	124.7	NI	[71]
*cis*-Carveol	*An. stephensi*	28.5	59.2	[72]
Eucalyptol	*An. anthropophagus*	>200	NI	[42]
(−)-Hydroxycarvone	*An. gambiae* s.s.	1172.2	NI	[71]
(−)-Isopulegol (2)	*An. gambiae* s.s.	49.4	NI	[71]
(+)-Limonene epoxide	*An. gambiae* s.s.	200.8	NI	[71]
(−)-Perillyl alcohol	*An. gambiae* s.s.	18.4	NI	[71]
Piperitenone oxide	*An. stephensi*	25.8	NI	[57]
Terpine-4-ol	*An. anthropophagus*	76.7	139.4	[42]
Terpinen-4-ol	*An. gambiae* s.s.	337.7	NI	[71]
Terpinen-4-ol	*An. subpictus*	43.3	84.1	[65]
Thymol	*An. stephensi*	48.88	NI	[68]
Thymol	*An. subpictus*	22.06	40.0	[48]
**Sesquiterpenes**				
α-Humulene	*An. subpictus*	6.19	12.0	[65]
β-Caryophyllene	*An. subpictus*	41.66	84.9	[65]
β-Elemene	*An. subpictus*	10.26	20.0	[65]
Caryophyllene	*An. anthropophagus*	>200		[42]
Germacrene D	*An. anthropophagus*	49.81	106.2	[42]
α-Cadinol	*An. subpictus*	10.27	20.4	[74]
Ar-turmerone	*An. quadrimaculatus*	2.8	7.0	[50]
Caryophyllene oxide	*An. anthropophagus*	49.46	115.4	[42]
Germacrene D-4-ol	*An. subpictus*	6.12	12.1	[74]
**Phenylpropenes**				
Eugenol	*An. subpictus*	25.45	50.6	[65]
**Others**				
Curcumin	*An. quadrimaculatus*	32.5	74.6	[50]
Desmethoxycurcumin	*An. quadrimaculatus*	29.7	66.7	[50]

LC_50_ and LC_90_ are the lethal concentrations that kill 50% and 90%, 95% and 99% of larvae, respectively.

**Table 4:** Essential oils’ toxicity against *Anopheles* sp. adults.

Plant Species	Plant Organ	Extraction Method	Mosquito	Methods	LC_50_ (mg/L)	LD_50_ (µg/g Mosquito)	LC_95_ (mg/mat)	LC_99_ (mg/L)	Ref.
* Deltamethrin			*An. gambiae*	TA		0.003			[76]
* Permethrin			*An. gambiae*	TA		0.6			[76]
*Apium graveolens* L.	NI	NI	*An. gambiae*	TA		600.0			[76]
*Apium graveolens* L.	NI	NI	*An. gambiae*	TA		4.5			[76]
*Apium graveolens* L.	NI	NI	*An. gambiae*	TA		18.0			[76]
*Apium graveolens* L.	seed	NI	*An. gambiae*	TA		6.6			[76]
*Artemisia absinthium*	NI	NI	*An. gambia*	TA		12.0			[76]
*Cedrus* sp.	NI	NI	*An. gambiae*	TA		3.8			[76]
*Chenopodium ambrosioides*	Leafy stems	Hydrod.	*An. gambiae*	WHOTK	0.9			2.1	[77]
*Cinnamomum zeylanicum*	Bark	Hydrod.	*An. stephensi*	MA			286.2		[36]
*Cinnamomun zeylanicum*	bark	NS	*An. gambiae*	TA		2.1			[76]
*Cinnamomun zeylanicum*	NI	NI	*An. gambiae*	TA		2.9			[76]
*Citrus sinensis*	NI	NI	*An. gambiae*	TA		11.1			[76]
*Cochlospermum planchonii*	Root	Hydrod.	*An. gambiae*	WHOTK	2.3			7.6	[77]
*Cuminum cyminum*	Seed	Hydrod.	*An. stephensi*	MA			305.2		[36]
*Curcuma longa*	Rhizome	Hydrod.	*An. stephensi*	MA			302.6		[36]
*Cymbopogon citratus*	Leaf	Hydrod.	*An. gambiae*	WHO test kits	0.2			0.4	[77]
*Cymbopogon citratus*	NI	NI	*An. gambiae*	TA		3			[76]
*Cymbopogon schoenanthus*	Leafy stems	Hydrod.	*An. gambiae*	WHOTK	1.57			2.7	[77]
*Cymbopogon winterianus*	NI	NI	*An. gambiae*	TA		3.9			[76]
*Eucalyptus tereticornis*	Leaf	Hydrod.	*An. gambiae*	WHOTK	0.148			1.4	[77]
*Gaultheria procumbens*	NI	NI	*An. gambiae*	TA		11.1			[76]
*Juniperus virginiana*	NI	NI	*An. gambiae*	TA		7.7			[76]
*Litsea cubeba*	NI	NI	*An. gambiae*	TA		4.0			[76]
*Mentha piperita*	NI	NI	*An. gambiae*	TA		6.8			[76]
*Myristica fragrans*	NI	NI	*An. gambiae*	TA		10.5			[76]
*Ocimum basilicum*	Leaf	Hydrod.	*An. stephensi*	MA			316.4		[36]
*Origanum vulgare*	NI	NI	*An. gambiae*	TA		1.6			[76]
*Pelargonium graveolens*	NI	NI	*An. gambiae*	TA		2.6			[76]
*Petroselinum crispum*	NI	NI	*An. gambiae*	TA		5.0			[76]
*Pimpinella anisum*	Seed	Hydrod.	*An. stephensi*	MA			378.5		[36]
*Piper nigrum*	NI	NI	*An. gambiae*	TA		8			[76]
*Rosmarinus officinalis*	NI	NI	*An. gambiae*	TA		31			[76]
*Rosmarinus officinalis*	Shoot	Hydrod.	*An. stephensi*	MA			282.6		[36]
*Sassafras* sp.	NI	NI	*An. gambiae*	TA		10			[76]
*Sesamum indicum*	NI	NI	*An. gambiae*	TA		5.9			[76]
*Syzygium aromaticum*	leaf	NI	*An. gambiae*	TA		1.5			[76]

* Chemical Positive controls; NI: Information is not available; TA = Topical application; MA = Mat machine, WHOTK = WHO test kits, LC_50_, LC_95_ and LC_99_ were Lethal Concentration that killed 50%, 95% and 99% adults mosquitoes LC_50_, LC_95_ and LC_99_ were Lethal Concentration that killed 50%, 95% and 99% adults mosquitoes LC_50_, LC_95_ and LC_99_ were Lethal Concentration that killed 50%, 95% and 99% adults mosquitoes, LD 50 was Lethal Dose that killed 50% of adults mosquitoes.

**Table 5:** Plant essential oils with high repellency to *Anopheles* species.

Plant Species	Plant Organ	Extraction Method	Moquito	Test Method	Concentration (mg/cm^2^)	Protection Time (mn)	% Repellency	RD_95_ (mg/mat)	Ref.
*Amyris balsamifera*	NI	NI	*An. stephensi*	Human bait	0.5	480.0	100.0		[81]
*Anethum graveolens*	NI	NI	*An. stephensi*	Human bait	0.5	210.0	71.4		[81]
*Aniba rosaeodora*	NI	NI	*An. stephensi*	Human bait	0.5	390.0	4.8		[81]
*Anthemis nobilis*	NI	NI	*An. stephensi*	Human bait	0.5	480.0	76.2		[81]
*Boswellia carteri*			*An. stephensi*	Human bait	0.5	300.0	19.0		[81]
*Cananga odorata*	*Flower*	*Steamd.*	*An. dirus*	Human bait	0.2	24.0	92.0		[84]
*Chamaemelum nobile*	NI	NI	*An. stephensi*	Human bait	0.5	330.0	47.6		[81]
*Cinnamomum camphora*	NI	NI	*An. stephensi*	Human bait	0.5	480.0	42.8		[81]
*Cinnamomum zeylanicum*	NI	NI	*An. stephensi*	Human bait	0.5	480.0	100.0		[81]
*Cinnamomum zeylanicum*	*Bark*	*Hydrod.*	*An. stephensi*	Cage test				49.6	[36]
*Citrus limon*	NI	NI	*An. stephensi*	Human bait	0.5	420.0	9.5		[81]
*Citrus sinensis*	*Fruit*	*Steamd.*	*An. dirus*	Human bait	0.21	24	84		[84]
*Curcuma longa*	*Rhizome*	*Hydrod.*	*An. stephensi*	Cage test				93.7	[36]
*Cymbopogon citratus*	*Leaf and stem*	*Steamd.*	*An. dirus*	Human bait	0.2	132.0	98.0		[84]
*Cymbopogon citratus*	NI	NI	*An. stephensi*	Human bait	0.5	480.0	100.0		[81]
*Cymbopogon nardus*	*Leaf*	*Steamd.*	*An. dirus*	Human bait	0.2	90.0	98.0		[84]
*Cymbopogon winterianus*	NI	NI	*An. stephensi*	Human bait	0.5	480.0	52.4		[81]
*DEET*	NI	NI	*An. stephensi*	Human bait	0.5	480.0	100.0		[81]
*Eucalyptus citriodora*	*Leaf*	*Steamd.*	*An. dirus*	Human bait	0.2	30.0	86.0		[84]
*Eucalyptus citriodora*	NI	NI	*An. stephensi*	Human bait	0.5	480.0	52.4		[81]
*Eucalyptus dives*	NI	NI	*An. stephensi*	Human bait	0.5	480.0	38.1		[81]
*Eucalyptus globulus*	NI	NI	*An. stephensi*	Human bait	0.5	330.0	28.6		[81]
*Eucalyptus radiata*	NI	NI	*An. stephensi*	Human bait	0.5	480.0	42.8		[81]
*Ferula galbaniflua*	NI	NI	*An. stephensi*	Human bait	0.5	480.0	100.0		[81]
*Glycina max*	NI	NI	*An. stephensi*	Human bait	0.5	480.0	76.2		[81]
*Glycina soja*	NI	NI	*An. stephensi*	Human bait	0.5	480.0	9.5		[81]
*Helichrysum italicum*	NI	NI	*An. stephensi*	Human bait	0.5	360.0	47.6		[81]
*Jasminum grandiflorum*	NI	NI	*An. stephensi*	Human bait	0.5	480.0	100.0		[81]
*Juniperus communis*	NI	NI	*An. stephensi*	Human bait	0.5	480.0	76.2		[81]
*Juniperus virginiana*	NI	NI	*An. stephensi*	Human bait	0.5	480.0	38.1		[81]
*Lavandula angustifolia*	NI	NI	*An. stephensi*	Human bait	0.5	480.0	80.9		[81]
*Lippia citriodora*	NI	NI	*An. stephensi*	Human bait	0.5	330.0	38.1		[81]
*Litsea cubeba*	NI	NI	*An. stephensi*	Human bait	0.5	480.0	100.0		[81]
*Melaleuca leucadendron*	NI	NI	*An. stephensi*	Human bait	0.5	480.0	100.0		[81]
*Melaleuca quinquenervia*	NI	NI	*An. stephensi*	Human bait	0.5	480.0	100.0		[81]
*Mentha piperita*	NI	NI	*An. stephensi*	Human bait	0.5	390.0	57.1		[81]
*Myrtus communis*	NI	NI	*An. stephensi*	Human bait	0.5	390.0	42.8		[81]
*Nepeta cataria*	NI	NI	*An. stephensi*	Human bait	0.5	480.0	100.0		[81]
*Ocimum basilicum*	*Leaf*	*Hydrod.*	*An. stephensi*	Cage test				75.0	[36]
*Ocimum basilicum*	*Leaf*	*Steamd.*	*An. dirus*	Human bait	0.2	96.0	96.0		[84]
*Ocimum basilicum*	NI	NI	*An. stephensi*	Human bait	0.5	210.0	66.7		[81]
*Olea europaea*	NI	NI	*An. stephensi*	Human bait	0.5	480.0	71.4		[81]
*Pelargonium graveolens*	NI	NI	*An. stephensi*	Human bait	0.5	480.0	61.9		[81]
*Picea excelsa*	NI	NI	*An. stephensi*	Human bait	0.5	180.0	19.0		[81]
*Pimpinella anisum*	*Seed*	*Hydrod.*	*An. stephensi*	Cage test				154.1	[36]
*Piper nigrum*	NI	NI	*An. stephensi*	Human bait	0.5	180.0	61.9		[81]
*Rosmarinus officinalis*	*Shoot*	*Hydrod.*	*An. stephensi*	Cage test				38.9	[36]
*Rosmarinus officinalis*	NI	NI	*An. stephensi*	Human bait	0.5	480.0	100.0		[81]
*Salvia sclarea*	NI	NI	*An. stephensi*	Human bait	0.5	300.0	19.0		[81]
*Santalum album*	NI	NI	*An. stephensi*	Human bait	0.5	480.0	100.0		[81]
*Syzygium aromaticum*	*Flower*	*Steamd.*	*An. dirus*	Human bait	0.2	96.0	98.0		[84]
*Tagetes minuta*	NI	NI	*An. stephensi*	Human bait	0.5	480.0	100.0		[81]
*Thymus serpyllum*	NI	NI	*An. stephensi*	Human bait	0.5	450.0	33.3		[81]
*Viola odorata*	NI	NI	*An. stephensi*	Human bait	0.5	480.0	100.0		[81]

DEET: *N*,*N*-diethyl-3-methylbenzanmide, used as repellent positive control; RD_50_ and RD_95_ are the doses that repel 50% and 95% of adults, respectively [44].

**Table 6:** Essential oil composition and their activity against Anophelineae populations.

Plant Species	Essential Oil Major Components (%)	Mono Hydro	Mono Oxy	Sesqui Hydro	Sesqui Oxy	Mosquito Species	Larvicidal LC_50_ (mg/L)	Adulcidal LC_50_ (mg/L)	Ref.
*Ocimum basilicum*	Linalool (52.4), Methyl eugenol (18.7)	9.9	79.8	13.4		*An. subpictus*	9.7		[56]
*Blumea densiflora*	Borneol (11.4), germacrene D. (8.66), β-caryophyllene (6.6), γ-terpinene (4.3), sabinene (4.3), and β-bisabolene (4.2)	25.9	25.2	29.3	10.7	*An. anthropophagus*	10.0		[42]
*Tagetes patula*	Limonene (13.60), terpinolene (11.2), *Z*-β-ocimene (8.3), *E*-caryophyllene (8.0), piperitone (6.1), *p*-cymen-8-ol (5.4), piperitenone (4.9)	40.6	21.2	9.3	3.1	*An. stephensi*	12.1		[66]
*Ferulago carduchorum*	(*Z*)-β-ocimene (43.3), α-pinene (18.2), bornyl acetate (3.9)	78.6	5.9	5.9	0.7	*An. stephensi*	12.8		[53]
*Chloroxylon swietenia*	Geijerene (26.9), limonene (15.2), germacrene D (10.6), pregeijerene (7.8)	22.9	2.7	53.9	3.0	*An. stephensi*	14.9		[77]
*Feronia limonia*	Estragole (34.7), β-pinene (23.6), (*Z*)-caryophyllene (11.0), methyl (*Z*)-caryophyllene (11.0).	32.5	46.9	13.0	7.2	*An. Stephensi*	15.0		[52]
*Cymbopogon citratus*	Geranial (39.3), neral (21.9), geraniol (15.6), myrcene (14.00)	15.8	81.6	0.5		*An. gambiae*	18.0		[38]
*Chloroxylon swietenia*	Limonene (12.9), geijerene (17.7), pregeijerene (9.92), germacrene D (8.84)	21.1	5.47	41.5	8.6	*An. stephensi*	19.0		[45]
*Bunium persicum*	*p*-Cuminaldehyde (23.5), α-methylbenzenemethanol (14.6), camphor (13.5), γ-terpinene (13.1), β-cymene (8.5)	33.3	53.7	5.8	1.5	*An. stephensi*	27.7		[43]
*Plectranthus amboinicus*	Carvacrol (28.6), thymol (21.7), α-humulene (9.7), undecanal (8.3)	15.1	64.2	11.7	8.6	*An. stephensi*	28.4		[62]
*Citrus aurantium*	d,l-limonene(94.8)	97.4	0.7	0.1	0.06	*A. stephensi*	31.2		[47]
*Plectranthus mollis*	Piperitone oxide (23.7), fenchone (19.2), piperitenone oxide (13.0), β-caryophyllene (10.3)	5.6	60.2	18.3	0.9	*An. stephensi*	33.5		[55]
*Cymbopogon citratus*	Myrcene (11.4), neral (30.2), geranial (32.8)	14.1	74.5	1.3	0.1	*An. funestus*	34.6		[51]
*Piper capense*	δ-Cadinene (16.8), β-pinene (7.2), β-bisabolene (5.6), α-phellandrene (4.7), myristicin (4.), α-pinene (3.9), sabinene (3.8), β-cubebene (3.3), bicyclogermacrene (3.3), limonene (3.1), β-phellandrene (2.5), linalool (2.4), spathulenol (2.4)	30.6	4.3	43.9	6.1	*An. gambiae*	34.9		[60]
*Zingiber nimmoni*	β-Caryophyllene (26.9), α-humulene (19.6), α-cadinol (5.20), myrcene (5.10)	19.7	9.5	51.9	16.2	*An. stephensi*	41.2		[59]
*Amomum subulatum*	1.8-cineole (39.8), α-terpineol (11.5), β-pinene (4.2), terpinen-4-ol (3.9)	50.7	23.7	9.0	11.0	*An. subpictus*	41.2		[40]
*Mentha spicata*	carvone (48.6), cis-carveol (21.3), limonene (11.3)	11.3	83.2	3.2	0.7	*An. stephensi*	49.7		[87]
*Artemisia gilvescens*	Camphor (13.5), eucalyptol (12.1), terpine-4-ol (9.6), germacrene D (8.6)	10.1	53.9	18.9	9.0	*An. anthropophagus*	49.9		[42]
*Plectranthus amboinicus*	Carvacrol (29.2), thymol (21.7), α-humulene (9.7), undecanal (8.3)	14.9	64.2	9.7	7.9	*An. gambiae*	55.2		[61]
*Murraya exotica*	β-Humulene (40.6), benzyl benzoate (23.9)	5.66	1.0	61.5	6.01	*An. stephensi*	56.3		[58]
*Zanthoxylum armatum*	Linalool (57.0), limonene (19.8), *E*-methyl cinnamate (5.7)	21.7	75.0			*An. stephensi*	58.0		[69]
*Coleus aromaticus*	thymol (82.6)	2.8	88.7	3.2	1.3	*An. subpictus*	60.3		[48]
*Zhumeria majdae*	Linalool (31.2), camphor (38.5)	18.9	77.4	0.6	0.7	*An. stephensi*	61.3		[70]
*Lavandula gibsoni*	Thymol (10.4), α-terpinolene (22.2)	5.6	60.2	18.3	0.9	*An. stephensi*	62.8		[55]
*Origanum vulgare*	Carvacrol (38.3), terpiene-4-ol (28.7)	7.7	80.6	5.2	3.7	*An. stephensi*	67.0		[88]
*Cryptomeria japonica*	Kau-16-rene (23.3), β-elemol (18.3)	28.9	6.5	0.4	39.0	*An. gambiae*	67.1		[49]
*Origanum vulgare*	Carvacrol (38.30), terpiene-4-ol (28.7)	7.7	80.6	5.2	3.7	*An. subpictus*	74.1		[88]
*Trachyspermum ammi*	Thymol (66.7), *p*-cymene (17.4), γ-terpenene (10.1)	29.8	67.9			*An. stephensi*	80.8		[68]
*Mentha spicata*	Piperitenone oxide (71.1), carvone (5.8), β-caryophyllene (2.3), limonene (1.3)	1.4	76.9	2.3		*An. stephensi*	82.9		[57]
*Syzygium zeylanicum*	α-Humulene (37.8), β-elemene (10.7)	1.2	0.9	64.6	24.9	*An. subpictus*	83.1		[65]
*Plectranthus barbatus*	Eugenol (31.1), α-pinene (19.4), β-caryophyllene (18.4).	25.2	31.1	38.9	2.6	*An. subpictus*	84.2		[89]
*Ocimum canum*	1.8-Cineole (29.4), linalool (19.1), Perpinen-4-ol (7.5)	10.4	35.2	8.4		*An. funestus*	91.2		[51]
*Eucalyptus tereticornis*	*p*-Cymene (16.7), caryophyllene oxide (14.2), spathulenol (13.5). cryptone (11.4)	23.9	20.0	2.0	34.7	*An. gambiae*		0.1	[77]
*Cymbopogon citratus*	Neral (33.1), geranial (44.3)	12.9	81.1	0.2	0.5	*An. gambiae*		0.2	[77]
*Chenopodium ambrosioides*	Ascaridole (41.9), α-terpinene (16.5), *p*-cymene (14.4), isoascridole (7.5)	31.6	51.5			*An.gambiae*		1.0	[44]
*Cymbopogon schoenanthus*	piperitone (58.9), δ-2-carene (15.5)	19.8	63.5	2.3	12.8	*An. gambiae*		1.6	[77]

Mono hydro: Monoterpenes hydrocarbon; Mono Oxy: Monoterpenes Oxygenated; Sesquiterpenes hydro: Sesquiterpenes hydrocarbon; Sesquiterpenes Hydro; Sesquiterpenes Oxygenated.

## Abbreviations

EOs	Essential Oils
IRS	Indoor residual spraying
LLINs	Long-lasting insecticide treated nets
*kdr*	*knock down resistance*
LD	Lethal dose
LC	Lethal concentration
US EPA	United States Environmental Protection Agency
WHO	World Health Organization
DEET	*N*,*N*-diethyl-3-methylbenzamide
AChE	Acetylcholinesterase
ACh	Acetylcholine
OA	Octopamine
GABA	Gamma aminobutyric acid

## References

[1] WHO (2015). World Malaria Report 2015.

[2] WHO (2012). Global Plan for Insect Management.

[3] Kelly-Hope L., Ranson H., Hemingway J. (2008). Lessons from the past: Managing insecticide resistance in malaria control and eradication programmes. Lancet Infect. Dis..

[4] Namountougou M., Diabaté A., Etang J., Bass C., Sawadogo S.P., Gnankinié O., Baldet T., Martin T., Chandre F., Simard F. (2013). First report of the L1014S kdr mutation in wild populations of *Anopheles gambiae* M and S molecular forms in Burkina Faso (West Africa). Acta Trop..

[5] Namountougou M., Frédéric S., Baldet T., Diabate A., Ouédraogo J.-B., Martin T., Dabire R.K. (2012). Multiple Insecticide Resistance in *Anopheles gambiae* s.l. Populations from Burkina Faso, West Africa. PLoS ONE.

[6] Gnankiné O., Bassolé I.H.N., Chandre F., Glitho I., Akogbeto M., Dabiré R.K., Martin T. (2013). Insecticide resistance in *Bemisia tabaci* Gennadius (Homoptera: Aleyrodidae) and *Anopheles gambiae* Giles (Diptera: Culicidae) could compromise the sustainability of malaria vector control strategies in West Africa. Acta Trop..

[7] Dabiré R., Namountougou M., Sawadogo S., Yaro L., Toé H., Ouari A., Gouagna L.-C., Simard F., Chandre F., Baldet T. (2012). Population dynamics of *Anopheles gambiae* s.l. in Bobo-Dioulasso city: bionomics, infection rate and susceptibility to insecticides. Parasites Vectors.

[8] Silva A.P.B., Santos J.M.M., Martins A.J. (2014). Mutations in the voltage-gated sodium channel gene of anophelines and their association with resistance to pyrethroids—A review. Parasites Vectors.

[9] Chandre F., Darrier F., Manga L., Akogbeto M., Faye O., Mouchet J., Guillet P. (1999). Status of pyrethroid resistance in *Anopheles gambiae* sensu lato. Bull. World Health Organ..

[10] Diabaté A., Baldet T., Chandre F., Guiguemde R., Brengues C., Guillet P., Hemingway J., Hougard J. (2002). First report of the kdr mutation in *Anopheles gambiae* M form from Burkina Faso, West Africa. Parassitologia.

[11] Awolola T.S., Oyewole I.O., Amajoh C.N., Idowu E.T., Ajayi M.B., Oduola A., Manafa O.U., Ibrahim K., Koekemoer L.L., Coetzee M. (2005). Distribution of the molecular forms of *Anopheles gambiae* and pyrethroid knock down resistance gene in Nigeria. Acta Trop..

[12] Fanello C., Santolamazza F., Della-Torre A. (2002). Simultaneous identification of species and molecular forms of the *Anopheles gambiae* complex by PCR-RFLP. Med. Vet. Entomol..

[13] Weill M., Chandre F., Brengues C., Manguin S., Akogbeto M., Pasteur N. (2000). The kdr mutation occurs in the Mopti form of *Anopheles gambiae* s.s. through introgression. Insect Mol. Bol..

[14] Della-Torre A., Fanello C., Akogbeto M., Favia G., Petrarca V., Coluzzi M. (2001). Molecular evidence of incipient speciation within *Anopheles gambiae* s.s. in West Africa. Insect Mol. Bol..

[15] N’Guessan R.N., Corbel V., Akogbéto M., Rowland M. (2007). Reduced Efficacy of Insecticide- treated Nets and Indoor Residual Spraying for Malaria Control in Pyrethroid Resistance Area, Benin. Emerg. Infect. Dis..

[16] Diabaté A., Baldet T., Chandre F., Akogbeto M., Guiguemde T., Darriet F., Brengues C., Small G., Hougard J. (2002). The role of agricultural use of insecticides in resistance to pyrethroids in *Anopheles gambiae* s.l. in Burkina Faso. Am. J. Trop. Med. Hyg..

[17] Verhaeghen K., Van Bortel W., Roelants P., Backeljau T., Coosemans M. (2006). Detection of the East and West African kdr mutation in *Anopheles gambiae* and *Anopheles arabiensis* from Uganda using a new assay based on FRET/Melt Curve analysis. Malar. J..

[18] Etang J., Fondjo E., Chandre F., Morlais I., Brengues C., Nwane P., Chouaibou M., Ndjemai H., Frédéric S. (2006). Short report: First report of knockdown mutations in the malaria vector *Anopheles gambiae* from cameroon. Am. J. Trop. Med. Hyg..

[19] Nwane P., Etang J., Chouaibou M., Toto J., Koffi A., Mimpfound R., Simard F. (2013). Multiple insecticide resistance mechanisms in *Anopheles gambiae* s.l. populations from Cameroon, Central Africa. Parasites Vectors.

[20] Djégbe I., Boussari O., Sidick A., Martin T., Ranson H., Chandre F., Akogbéto M., Corbel V. (2011). Dynamics of insecticide resistance in malaria vectors in Benin: First evidence of the presence of L1014S kdr mutation in *Anopheles gambiae* from West Africa. Malar. J..

[21] Padonou G., Sezonlin M., Ossè R., Aïzoun N., Agbo F., Oussou O., Gbédjissi G., Akogbéto M. (2012). Impact of three years of large scale indoor residual spraying (IRS) and insecticide treated nets (ITNs) interventions on insecticide resistance in *Anopheles gambiae* s.l. in Benin. Parasites Vectors.

[22] Zoubiri S., Baaliouamer A. (2014). Potentiality of plants as source of insecticide principles. J. Saudi Chem. Soc..

[23] Tripathi A.K., Upadhyay S., Bhuiyan M., Bhattacharya P.R. (2009). A review on prospects of essential oils as biopesticide in insect-pest management. J. Pharmacogn. Phyther..

[24] Isman M.B., Miresmailli S., Machial C. (2011). Commercial opportunities for pesticides based on plant essential oils in agriculture, industry and consumer products. Phytochem. Rev..

[25] Rehman J.U., Ali A., Khan I.A. (2014). Fitoterapia Plant based products: Use and development as repellents against mosquitoes: A review. Fitoterapia.

[26] Gnankiné O. (2012). Use of biopesticides in the perspective of chemical resistance management in Western Africa: The cases of Bemisia tabaci (Homotera: Aleyrodidae) and *Anopheles gambiae* (Diptera: Culicidae). Trends Entomol..

[27] Nerio S.L., Olivero-verbel J., Stashenko E. (2010). Bioresource Technology Repellent activity of essential oils: A review. Bioresour. Technol..

[28] Katz T., Miller J.H., Hebert A. (2008). Insects repellents: Historical perspectives and new developments. J. Am. Acad. Dermatol..

[29] Guenther E. (1948). The Essential Oils.

[30] ASTA (1968). Official Analytical Methods of the American Spice Trade Association.

[31] Chialva F., Gabri G., Liddle P.A.P., Ulian F. (1982). Qualitative evaluation of aromatic herbs by direct headspace GC analysis. Application of the method and comparison with the traditional analysis of essential oil. J. High Resolut. Chromatogr..

[32] Burbott A.J., Loomis W.D. (1967). Effects of light and temperature on the monoterpenes of peppermint. Plant Physiol..

[33] Takeoka G., Ebeler S., Jennings W. (1985). Capillary gas chromatographic analysis of volatile flavor compounds. American Chemical Society Symposium.

[34] Bakkali F., Averbeck S., Averbeck D., Idaomar M. (2008). Biological effects of essential oils—A review. Food Chem. Toxicol..

[35] Glasby J. (1982). Encyclopédia of the Terpenoids.

[36] Prajapati V., Tripathi A.K., Aggarwal K.K., Khanuja S.P.S. (2005). Insecticidal, repellent and oviposition-deterrent activity of selected essential oils against *Anopheles stephensi*, *Aedes aegypti* and *Culex quinquefasciatus*. Bioresour. Technol..

[37] Bassolé I., Guelbeogo W., Nébié R., Costantini C., Sagnon N., Kaboré Z., Traoré S. (2003). Ovicidal and larvicidal activity against Aedes aegypti and *Anopheles gambiae* complex mosquitoes of essential oils extracted from three spontaneous plants of Burkina Faso. Parassitologia.

[38] Tchoumbougnang F., Dongmo P., Sameza L., Mbanjo N., Fotso G., Zollo P., Menut C. (2009). Activité larvicide sur *Anopheles gambiae* Giles et composition chimique des huiles essentielles extraites de quatre plantes cultivées au Cameroun. Biotechnol. Agron. Soc. Environ..

[39] Deletre E., Martin T., Campagne P., Bourguet D., Cadin A., Menut C., Bonafos R., Chandre F. (2013). Repellent, Irritant and Toxic Effects of 20 Plant Extracts on Adults of the Malaria Vector *Anopheles gambiae* Mosquito. PLoS ONE.

[40] Govindarajan M., Rajeswary M., Senthilmurugan S., Vijayan P., Alharbi N.S., Km S., Khaled J.M., Benelli G. (2017). Larvicidal activity of the essential oil from *Amomum subulatum* Roxb.(Zingiberaceae) against *Anopheles subpictus*, Aedes albopictus and *Culex tritaeniorhynchus* (Diptera: Culicidae), and non-target impact on four mosquito natural enemies. Physiol. Mol. Plant Pathol..

[41] Pitasawat B., Champakaew D., Choochote W., Jitpakdi A., Chaithong U. (2007). Aromatic plant-derived essential oil: An alternative larvicide for mosquito control. Fitoterapia.

[42] Zhu L., Tian Y. (2013). Chemical composition and larvicidal activity of essential oil of *Artemisia gilvescens* against *Anopheles anthropophagus*. Parsitol. Res..

[43] Sanei-dehkordi A., Vatandoost H., Abaei M.R. (2016). Chemical Composition and Larvicidal Activity of *Bunium persicum* Essential Oil Against Two Important Mosquitoes Vectors. J. Essen. Oil Bear. Plants.

[44] Massebo F., Tadesse M., Bekele T., Balkew M., Gebre-michael T. (2009). Evaluation on larvicidal effects of essential oils of some local plants against *Anopheles arabiensis* Patton and *Aedes aegypti* Linnaeus (Diptera, Culicidae) in Ethiopia. Afr. J. Biotechnol..

[45] Kiran S.R., Bhavani K., Devi P.S., Rao B.R.R., Reddy K.J. (2006). Composition and larvicidal activity of leaves and stem essential oils of *Chloroxylon swietenia* DC against *Aedes aegypti* and *Anopheles stephensi*. Bioresour. Technol..

[46] Mozaffari E., Abai M.R., Khanavi M., Vatandoost H., Sedaghat M.M., Moridnia A., Saber-Navaei M., Sanei-Dehkordi A., Rafi F. (2014). Chemical composition, larvicidal and repellency properties of *Cionura erecta* (L.) Griseb. against malaria vector, *Anopheles stephensi* liston (Diptera: Culicidae). J. Arthropod-Borne Dis..

[47] Sanei-dehkordi A., Sedaghat M.M., Vatandoost H. (2016). Original Article Chemical Compositions of the Peel Essential Oil of Citrus aurantium and Its Natural Larvicidal Activity against the Malaria Vector *Anopheles stephensi* (Diptera: Culicidae) in Comparison with *Citrus paradisi*. J. Arthropod-Borne Dis..

[48] Govindarajan M., Sivakumar R. (2013). Mosquito larvicidal activity of thymol from essential oil of *Coleus aromaticus* Benth. against *Culex tritaeniorhynchus*, *Aedes albopictus*, and *Anopheles subpictus* (Diptera: Culicidae). Parasitol. Res..

[49] Mdoe F.P., Cheng S., Lyaruu L., Nkwengulila G., Chang S., Kweka E.J. (2014). Larvicidal efficacy of *Cryptomeria japonica* leaf essential oils against *Anopheles gambiae*. Parasites Vectors.

[50] Ali A., Wang Y., Khan I.A. (2015). Larvicidal and Biting Deterrent Activity of Essential Oils of *Curcuma longa*, Ar-turmerone, and Curcuminoids Against *Aedes aegypti* and *Anopheles quadrimaculatus* (Culicidae: Diptera). J. Med. Entomol..

[51] Ntonga P.A., Baldovini N., Mouray E., Mambu L., Belong P., Grellier P. (2014). Activity of *Ocimum basilicum*, *Ocimum canum*, and *Cymbopogon citratus* essential oils against *Plasmodium falciparum* and mature-stage larvae of *Anopheles funestus* s. s. Parasite.

[52] Senthilkumar A., Jayaraman M., Venkatesalu V. (2013). Chemical constituents and larvicidal potential of *Feronia limonia* leaf essential oil against *Anopheles stephensi*, *Aedes aegypti* and *Culex quinquefasciatus*. Parsitol. Res..

[53] Golfakhrabadi F., Khanavi M., Ostad S.N., Saeidnia S. (2015). Original Article Biological Activities and Composition of Ferulago carduchorum Essential Oil. J. Arthropod Borne Dis..

[54] Karunamoorthi K., Girmay A., Hayleeyesus S.F. (2014). Mosquito repellent activity of essential oil of *Ethiopian ethnomedicinal* plant against Afro-tropical malarial vector *Anopheles arabiensis*. J. King Saud Univ. Sci..

[55] Kulkarni R.R., Pawar P.V., Joseph M.P., Akulwad A.K., Sen A., Joshi S.P. (2013). *Lavandula gibsoni* and *Plectranthus mollis* essential oils: Chemical analysis and insect control activities against *Aedes aegypti*, *Anopheles stephensi* and *Culex quinquefasciatus*. J. Pest Sci..

[56] Govindarajan M., Sivakumar R., Rajeswary M., Yogalakshmi K. (2013). Experimental Parasitology Chemical composition and larvicidal activity of essential oil from *Ocimum basilicum* (L.) against *Culex tritaeniorhynchus*, *Aedes albopictus* and *Anopheles subpictus* (Diptera: Culicidae). Exp. Parasitol..

[57] Tripathi A.K., Prajapati V., Ahmad A., Aggarwal K.K., Khanuja S.P.S. (2004). Piperitenone Oxide as Toxic, Repellent, and Reproduction Retardant Toward Malarial Vector *Anopheles stephensi* (Diptera: Anophelinae) *J*. Med. Entomol..

[58] Krishnamoorthy S., Chandrasekaran M. (2015). Identification of chemical constituents and larvicidal activity of essential oil from *Murraya exotica* L. (Rutaceae) against *Aedes aegypti*, *Anopheles stephensi* and *Culex quinquefasciatus* (Diptera: Culicidae). Parasitol. Res..

[59] Govindarajan M., Rajeswary M., Arivoli S. (2016). Larvicidal and repellent potential of *Zingiber nimmonii* (J. Graham) Dalzell (Zingiberaceae) essential oil: An eco-friendly tool against malaria, dengue, and lymphatic filariasis mosquito vectors?. Parasitol. Res..

[60] Matasyoh J.C., Wathuta E.M., Kariuki S.T., Chepkorir R. (2011). Journal of Asia-Paci fi c Entomology Chemical composition and larvicidal activity of *Piper capense* essential oil against the malaria vector, *Anopheles gambiae*. J. Asia Pac. Entomol..

[61] Kweka J.E., Senthilkumar A., Venkatesalu V. (2012). Toxicity of essential oil from Indian borage on the larvae of the African malaria vector mosquito, *Anopheles gambiae*. Parasites Vectors.

[62] Senthilkumar A., Venkatesalu V. (2010). Chemical composition and larvicidal activity of the essential oil of Plectranthus amboinicus (Lour.) Spreng against Anopheles stephensi: a malarial vector mosquito. Parsitol. Res..

[63] Govindarajan M., Rajeswary M., Veerakumar K., Muthukumaran U., Hoti S.L., Mehlhorn H., Barnard D.R. (2015). Novel synthesis of silver nanoparticles using *Bauhinia variegata*: A recent eco-friendly approach for mosquito control. Parasitol. Res..

[64] Ali A., Demirci B., Kiyan H.T., Bernier U.R., Tsikolia M., Wedge D.E., Khan I.A., Husnu K., Bas C.A.N. (2013). Biting Deterrence, Repellency, and Larvicidal Activity of *Ruta chalepensis* (Sapindales: Rutaceae) Essential Oil and Its Major Individual Constituents Against Mosquitoes. J. Med. Entomol..

[65] Govindarajan M., Benelli G. (2016). α-Humulene and β-elemene from *Syzygium zeylanicum* (Myrtaceae) essential oil: Highly effective and eco-friendly larvicides against *Anopheles subpictus*, *Aedes albopictus*, and *Culex tritaeniorhynchus* (Diptera: Culicidae). Parasitol. Res..

[66] Dharmagadda V.S.S., Naik S.N., Mittal P.K., Vasudevan P. (2005). Larvicidal activity of *Tagetes patula* essential oil against three mosquito species. Bioresour. Technol..

[67] Liu X.C., Dong H.W., Zhou L., Du S.S., Liu Z.L. (2013). Essential oil composition and larvicidal activity of *Toddalia asiatica* roots against the mosquito *Aedes albopictus* (Diptera: Culicidae). Parasitol. Res..

[68] Pandey S.K., Upadhyay S., Tripathi A.K. (2009). Insecticidal and repellent activities of thymol from the essential oil of *Trachyspermum ammi* (Linn) Sprague seeds against *Anopheles stephensi*. Parasitol. Res..

[69] Tiwary M., Naik S.N., Tewary D.K., Mittal P.K., Yadav S. (2007). Chemical composition and larvicidal activities of the essential oil of *Zanthoxylum armatum* DC (Rutaceae) against three mosquito vectors. J. Vector Borne Dis..

[70] Sanei-dehkordi A., Soleimani-ahmadi M., Akbarzadeh K., Abadi Y.S., Paksa A., Gorouhi M.A. (2016). Chemical Composition and Mosquito Larvicidal Properties of Essential Oil from Leaves of an Iranian Indigenous Plant *Zhumeria majdae*. J. Essen. Oil Bear. Plant..

[71] Kweka E.J., Lima T.C., Marciale C.M., Sousa D.P. (2016). De Asian Paci fi c Journal of Tropical Biomedicine. Asian Pac. J. Trop. Biomed..

[72] Govindarajan M., Mathivanan T., Elumalai K. (2011). Mosquito larvicidal, ovicidal, and repellent properties of botanical extracts against *Anopheles stephensi*, *Aedes aegypti*, and *Culex quinquefasciatus* (Diptera: Culicidae). Parsitol. Res..

[73] Govindarajan M., Benelli G. (2015). Facile biosynthesis of silver nanoparticles using *Barleria cristata*: *Mosquitocidal potential* and biotoxicity on three non-target aquatic organisms. Parsitol. Res..

[74] Pavela R., Pavela R. (2016). Essential oils for the development of eco-friendly mosquito larvicides: A review. Ind. Crop. Prod..

[75] Dias C.N., Moraes F.D.C. (2014). Essential oils and their compounds as Aedes aegypti L. (Diptera: Culicidae) larvicides: review. Parsitol. Res..

[76] Norris E.J., Gross A.D., Dunphy B.M., Bessette S. (2015). Comparison of the Insecticidal Characteristics of Commercially Available Plant Essential Oils Against *Aedes aegypti* and *Anopheles gambiae* (Diptera: Culicidae). J. Med. Entomol..

[77] Bossou A.D., Mangelinckx S., Yedomonhan H., Boko P.M., Akogbeto M.C., De Kimpe N., Avlessi F., Sohounhloue D.C.K. (2013). Chemical composition and insecticidal activity of plant essential oils from Benin against *Anopheles gambiae* (Giles). Parasites Vectors.

[78] WHO (2013). Test Procedures.

[79] Choochote W., Chaithong U., Kamsuk K., Jitpakdi A., Tippawangkosol P., Tuetun B., Champakaew D., Pitasawat B. (2007). Repellent activity of selected essential oils against *Aedes aegypti*. Fitoterapia.

[80] Deletre E., Schatz B., Chandre F., Ratnadass A. (2016). Prospects for repellent in pest control: Current developments and future challenges. Chemoecology.

[81] Amer A., Mehlhorn H. (2006). Repellency effect of forty-one essential oils against *Aedes*, *Anopheles*, and *Culex mosquitoes*. Parasitol. Res..

[82] WHO (1996). Report of the Who Informal Consultation on the Evaluation and Testing of Insecticides CTD/WHOPES/IC/96.1 Geneva: Control of Tropical Diseases.

[83] Rajikumar S., Jebanessan A. (2007). Repellent activity of selected plant essential oils against the malarial fever mosquito *Anopheles stephensi*. Trop. Biomed..

[84] Phasomkusolsil S., Soonwera M. (2011). Comparative mosquito repellency of essential oils against *Aedes aegypti* (Linn.), *Anopheles dirus* (Peyton and Harrison) and *Culex quinquefasciatus* (Say). Asian Pac. J. Trop. Biomed..

[85] Abagli A.Z., Alavo T.B.C. (2011). Essential Oil from Bush Mint, Hyptis suaveolens, is as Effective as DEET for Personal Protection against *Mosquito Bites*. Open Entomol..

[86] Ipek E., Zeytinoglu H., Okay S., Tuylu B.A., Kurkcuoglu M., Baser K.H.C. (2005). Food Chemistry Genotoxicity and antigenotoxicity of *Origanum oil* and carvacrol evaluated by Ames Salmonella/microsomal test. Food Chem..

[87] Govindarajan M., Sivakumar R., Rajeswari M. (2012). Chemical composition and larvicidal activity of essential oil from *Mentha spicata* (Linn.) against three mosquito species. Parasitol. Res..

[88] Govindarajan M., Rajeswary M., Hoti S.L., Benelli G. (2016). Research in Veterinary Science Larvicidal potential of carvacrol and terpinen-4-ol from the essential oil of *Origanum vulgare* (Lamiaceae) against *Anopheles stephensi*, *Anopheles subpictus*, *Culex quinquefasciatus* and *Culex tritaeniorhynchus* (Diptera: Culicidae). Res. Vet. Sci..

[89] Govindarajan M., Rajeswary M., Hoti S.L., Bhattacharyya A., Benelli G. (2016). Eugenol, α-pinene and β-caryophyllene from Plectranthus barbatus essential oil as eco-friendly larvicides against malaria, dengue and Japanese encephalitis mosquito vectors. Parasitol. Res..

[90] Deletre E., Chandre F., Williams L., Duménil C., Menut C., Martin T. (2015). Electrophysiological and behavioral characterization of bioactive compounds of the *Thymus vulgaris*, *Cymbopogon winterianus*, *Cuminum cyminum* and *Cinnamomum zeylanicum* essential oils against *Anopheles gambiae* and prospects for their use as bednet treatmen. Parasites Vectors.

[91] Jaenson T.G.T., Pålsson K. (2006). Borg-karlson Anna-Karin Evaluation of Extracts and Oils of Mosquito (Diptera: Culicidae) Repellent Plants from Sweden and Guinea-Bissau. J. Med. Entomol..

[92] Sukumar K., Perich M., Boobar L. (1991). Botanical derivatives in mosquito control: A review. J. Am. Mosq. Control Assoc..

[93] Jantan I., Zaki Z. (1999). Development of environment-friendly insect repellents from the leaf oils of selected malaysian plants. Rev. Biodivers. Environ. Conserv..

[94] Park B.-S.P., Choi W.-S., Kim J.-H., Kim K.-H., Lee S.-E. (2005). Monoterpenes from thyme (*Thymus vulgaris*) as potential mosquito repellents potential mosquito repellents. Source J. Am. Mosq. Control Assoc..

[95] Omolo M.O., Okinyo D., Ndiege I.O., Hassanali A. (2004). Repellency of essential oils of some Kenyan plants against *Anopheles gambiae*. Phytochemistry.

[96] Trongtokit Y., Rongsriyam Y., Komalamisra N. (2005). Comparative Repellency of 38 Essential Oils against Mosquito Bites. Phyther. Res..

[97] Gillij Y.G., Gleiser R.M., Zygadlo J.A. (2008). Mosquito repellent activity of essential oils of aromatic plants growing in Argentina. Bioresour. Technol..

[98] Odalo J.O., Omolo M.O., Malebo H., Angira J., Njeru P.M., Ndiege I.O., Hassanali A. (2005). Repellency of essential oils of some plants from the Kenyan coast against *Anopheles gambiae*. Acta Trop..

[99] Pridham J. (1967). Terpenoids in Plants.

[100] Davidson P., Parish M. (1989). Methods for testing the efficacy of food antimicrobials. Food Technol..

[101] Abbassy M., Abdelgaleil S., Rabie R. (2009). Insecticidal and synergistic effects of *Majorana hortensis* essential oil and some of its major constituents. Entomol. Exp. Appl..

[102] Berenbaum M., Neal J. (1985). Synergism between myristicin and xanthotoxin, a naturally cooccurring plant toxicant. J. Chem. Ecol..

[103] Regnault-Roger C., Vincent C., Arnason J.T. (2012). Essential Oils in Insect Control: Low-Risk Products in a High-Stakes World. Annu. Rev. Entomol..

[104] Prates H.T., Santos J.P., Waquil J.M., Fabris J.D., Oliveira A.B., Foster J.E., Lagoas S., Horizonte B. (1998). Insecticidal Activity of Monoterpenes Against *Rhyzopertha dominica* (F.) and *Tribolium castaneum* (Herbst). J. Stored Prod. Res..

[105] Regnault-Roger C. (1997). The potential of botanical essential oils for insect pest control. Integr. Pest Manag. Rev..

[106] Rattan R. (2010). Mechanism of action of insecticidal secondary metabolites of plant origin. Crop Prot..

[107] Fournier D., Mutero A. (1994). Modification of acetylcholinesterase as a mechanism of resistance to insecticide. Comp. Biochem. Physiol. C Pharmacol. Toxicol. Endocrinol..

[108] Aygun D., Doganay Z., Altintop L., Guven H., Onar M., Deniz T., Sunter T. (2002). Serum acetylcholinesterase and prognosis of acute organophosphate poisoning. J. Toxicol. Clin. Toxicol..

[109] Houghton P., Ren Y., Howes M. (2006). Acetylcholinesterase inhibitors from plants and fungi. Nat. Prod. Rep..

[110] Abdelgaleil S.A., Mohamed M.I., Badawy M.E., El-arami S.A. (2009). Fumigant and contact toxicities of monoterpenes to *Sitophilus oryzae* (L.) and *Tribolium castaneum* (Herbst) and their inhibitory effects on acetylcholinesterase activity. J. Chem. Ecol..

[111] Hideyuki T., Mitsuo M. (2001). Inhibition of acetylcholinesterase activity by essential oil from Bergamot. Koryo Terupen oyobi Seiyu Kagaku ni Kansuru Toronkai Koen Yoshishu.

[112] Seo S.M., Jung C.S., Kang J., Lee H.R., Kim S.W., Hyun J., Park I.K. (2015). Larvicidal and Acetylcholinesterase Inhibitory Activities of Apiaceae Plant Essential Oils and Their Constituents against *Aedes albopictus* and Formulation Development. J. Agric. Food Chem..

[113] Savelev S., Okello E., Perry N., Wilkins R., Perry E. (2003). Synergistic and antagonistic interactions of anticholinesterase terpenoids in *Salvia lavandulae* folia essential oil. Pharmacol. Biochem. Behav..

[114] Keane S., Ryan M. (1999). Purification, characterisation, and inhibition bymonoterpenes of acetylcholinesterase from the waxmoth, *Galleria mellonella* (L.). Insect Biochem. Mol. Biol..

[115] Evans P. (1981). Multiple receptor types for octopamine in the locust. J. Physiol..

[116] Evans P. (1984). Studies on the mode of action of octopamine, 5-hydroxytryptamine and proctolin on a myogenic rhythm in the locust. J. Exp. Biol..

[117] Enan E. (2001). Insecticidal activity of essential oils: Octopaminergic sites of action. Comp. Biochem. Physiol..

[118] Kostyukovsky M., Rafaeli A., Gileadi C., Demchenko N., Shaaya E. (2002). Activation of octopaminergic receptors by essential oil constituents isolated from aromatic plants: Possible mode of action against insect pests. Pest Manag. Sci..

[119] Evans P., Robb S. (1993). Octopamine receptor subtypes and their modes of action. Neurochem. Res..

[120] Howell K., Evans P. (1998). The characterization of presynaptic octopamine receptors modulating octopamine release from an identified neurone in the locust. J. Exp. Biol..

[121] Bloomquist J.R. (2003). Chloride channels as tools for developing selective insecticides. Arch. Insect Biochem. Physiol..

[122] Tong F., Coats J.R. (2010). Effects of monoterpenoid insecticides on [^3^H]-TBOB binding in house fly GABA receptor and ^36^Cl^−^ À uptake in American cockroach ventral nerve cord. Pestic. Biochem. Physiol..

[123] Hold M., Sirisoma S., Ikeda T., Narahashi T., Casida E. (2000). Thujone (the active component of absinthe): aminobutyric acid type. A receptor modulation and metabolic detoxification. Proc. Natl. Acad. Sci. USA.

[124] Ratra G., Casida J. (2001). GABA receptor subunit composition relative to insecticide potency and selectivity. Toxicol. Lett..

[125] Priestley C., Williamson E., Wafford K., Satelle D.B. (2003). Thymol, a constituent of thyme essential oil, is a positive allosteric modulator of human GABA receptors and a homo-oligomeric GABA receptor from *Drosophila melanogaster*. Br. J. Pharmacol..

[126] Krimer V., Vaštag Ž., Radulovi L., Peri I., Preedy V. (2016). Microencapsulation Technology and Essential Oil Pesticides for Food Plant Production. Essential Oils in Food Preservation, Flavor and Safety.

[127] Devi N., Maji T.K., Stoytcheva M. (2011). Neem seed oil: Encapsulation and controlled release—Search for a greener alternative for pest control. Pesticides in the Modern World—Pesticides Use and Management.

[128] Maji T., Baruah I., Dube S., Hussain M. (2007). Microencapsulation of *Zanthoxylum limonella oil* (ZLO) in glutaraldehyde crosslinked gelatin for mosquito repellent application. Bioresour. Technol..

[129] Tawatsin A., Wratten S.D., Scott R.R., Thavara U., Techadamrongsin Y. (2001). Repellency of volatile oils from plants against three mosquito vectors. J. Vector Ecol..

[130] Specos M.M., Garcia J.J., Tornesello J., Marino P., Vecchia M.D., Tesoriero M.D., Hermida L.G. (2010). Transactions of the Royal Society of Tropical Medicine and Hygiene Microencapsulated citronella oil for mosquito repellent finishing of cotton textiles. Trans. R. Soc. Trop. Med. Hyg..

[131] Solomon B., Sahle F., Gebre-Mariam T., Asres K., Neubert R. (2012). Microencapsulation of citronella oil for mosquito-repellent application: Formulation and in vitro permeation studies. Eur. J. Pharm. Biopharm..

[132] Sakulkua U., Nuchuchuaa O., Uawongyartb N., Puttipipatkhachornc S., Soottitantawatd A., Ruktanonchaia U. (2009). Characterization and mosquito repellent activity of citronella oil nanoemulsion. Int. J. Pharm. Nanotechnol..

[133] Duvallet G., De Gentile L. (2012). Protection Personnelle Antivectorielle.

[134] Zaim M., Aitio A., Nakashima N. (2000). Safety of pyrethroid-treated mosquito nets. Med. Vet. Entomol..

[135] Palchick S. (1996). The Biology of Disease of Disease Vectors.

[136] McAllister C., Adams M.F. (2010). Mode of Action for Natural Products Isolated From Essential Oils of Two Trees Is Different From Available Mosquito Adulticides. J. Med. Entomol..

[137] Chitwood D. (2002). Phytochemical based strategies for nematode control. Annu. Rev. Phytopathol..

[138] Yadouleton A., Martin T., Padonou G., Chandre F., Asidi A., Djogbenou L., Dabiré R., Aïkpon R., Boko M., Glitho I. (2011). Cotton pest management practices and the selection of pyrethroid resistance in *Anopheles gambiae* population in Northern Benin. Parasites Vectors.

